# Effect of prednisolone therapy on serum levels of 1,2‐*O*‐dilauryl‐*rac*‐glycero glutaric acid‐(6′‐methylresorufin) ester lipase in dogs

**DOI:** 10.1111/jvim.15946

**Published:** 2020-11-04

**Authors:** Beatriz Mendoza, Maria Joana Dias, Telmo Nunes, Maria Alexandra Basso, Juan Hernandez, Rodolfo Oliveira Leal

**Affiliations:** ^1^ Faculdade de Medicina Veterinária Universidade de Lisboa Lisbon Portugal; ^2^ Internal Medicine Service ONIRIS, Ecole Nationale Vétérinaire Nantes France; ^3^ Centro de Investigação Interdisciplinar em Sanidade Animal (CIISA), Faculdade de Medicina Veterinária Universidade de Lisboa Lisbon Portugal

**Keywords:** corticosteroids, dog, laboratory diagnosis, pancreatitis

## Abstract

**Background:**

Activity of 1,2‐*O*‐dilauryl‐*rac*‐glycero glutaric acid‐(6′‐methylresorufin) ester (DGGR) lipase in serum shows good agreement with pancreatic lipase (cPL) in dogs. Although prednisolone therapy does not seem to affect serum cPL concentration, its influence on DGGR lipase is unclear.

**Objectives:**

The aim of the study was to evaluate the effect of prednisolone therapy on DGGR lipase serum activity in dogs.

**Animals:**

Thirty‐four dogs were used, of which 17 dogs received prednisolone (study group) and 17 healthy dogs did not receive treatment.

**Methods:**

A prospective cohort study measured DGGR lipase activity in both groups at 3 time points: T0, T1, and T2, corresponding to days 0, 7‐10, and 21‐30, respectively. Dogs from study group presented a medical reason that justified the use of prednisolone for at least 3 weeks. Initial prednisolone dose was .5‐2.0 mg/kg/day PO with a reduction at T1 to a final dose that was maintained until T2. DGGR lipase activity >160 U/L was defined as clinically relevant.

**Results:**

In the study group, DGGR lipase activity increased significantly from T0 to T1 (*P* = .02) and decreased significantly from T1 to T2 (*P* = .02). Median DGGR activity at each time point (T0, T1, and T2) was 24.74 (14.45–31.48), 36.82 (23.8–80.16), and 29.52 (15.91–48.48) U/L, respectively. In the control group, no significant changes were observed over time (*P* = .93). The DGGR lipase activity and prednisolone doses were not correlated for both T0‐T1 (*r*_s_ = .371, *P* = .14) and T1‐T2 (*r*_s_ = 0.390, *P* = .12).

**Conclusion and Clinical Importance:**

DGGR lipase activity was affected by prednisolone administered orally in dogs. However, this variation was not clinically important as values remained below the relevant upper limit.

**ABBREVIATIONS**
1,2 DiG: 1,2‐dyglyceride
DGGR: 1,2‐*O*‐dilauryl‐*rac*‐glycero glutaric acid‐(6′‐methylresorufin) ester
mRNA: messenger ribonucleic acid
Spec cPL: specific canine pancreatic lipase

## 1 INTRODUCTION

Digestive enzymes are the most commonly used biomarkers of pancreatic disease.^1^, ^2^, ^3^ Despite continuous progress in clinical research toward the development of a better pancreatic biomarker, no blood test is 100% accurate for the diagnosis of pancreatitis.^4^ Specific canine pancreatic lipase (Spec cPL) measurement is considered the most sensitive and specific noninvasive test for the diagnosis of pancreatitis.^5^, ^6^, ^7^ A serum lipase activity assay using the 1,2‐*O*‐dilauryl‐*rac*‐glycero glutaric acid‐(6′‐methylresorufin) ester (DGGR) substrate is more specific than the traditional 1,2‐diglyceride (1,2 DiG) substrate and as sensitive as Spec cPL.^4^, ^8^, ^9^ There is a high level of agreement between DGGR lipase and Spec cPL assays.^8^, ^10^ DGGR lipase has the advantage of being less expensive, with a shorter analysis protocol that allows rapid reporting of results.^10^ Since its validation for use in dogs, DGGR‐based lipase is considered acceptable for inclusion in routine chemistry panels as a screening biomarker for pancreatitis in dogs.^9^

Influence of endogenous hypercortisolemia on DGGR lipase is reported in dogs with naturally occurring hyperadrenocorticism.^11^, ^12^ However, the influence of exogenous corticosteroids on this biomarker remains unclear. Prednisolone or prednisone therapy does not affect serum canine pancreatic lipase immunoreactivity (cPLI) measurements in healthy dogs and dogs with X‐linked hereditary nephritis.^13^, ^14^ However, increased cPLI values in dogs with immune‐mediated diseases treated with prednisolone are reported.^15^ Glucocorticoids are frequently used drugs in veterinary medicine, and prednisolone is one of the most prescribed glucocorticoid compounds in clinical veterinary practice.^16^ However, it is unclear whether DGGR lipase values are affected by exogenous steroid therapy.

This study aimed to evaluate the influence of prednisolone therapy on DGGR lipase activity. It is hypothesized that the effect of prednisolone (0.5‐2.0 mg/kg/day) is not clinically relevant for DGGR lipase activity.

## 2 MATERIALS AND METHODS

### 2.1 Sample population

Thirty‐four client‐owned dogs were prospectively enrolled in the study. The dogs were recruited at the Veterinary Teaching Hospital—Faculty of Veterinary Medicine—University of Lisbon from August 2019 to February 2020. Of the 34 enrolled animals, 17 each were enrolled in the study group (SG) and control group (CG).

**FIGURE 1:**
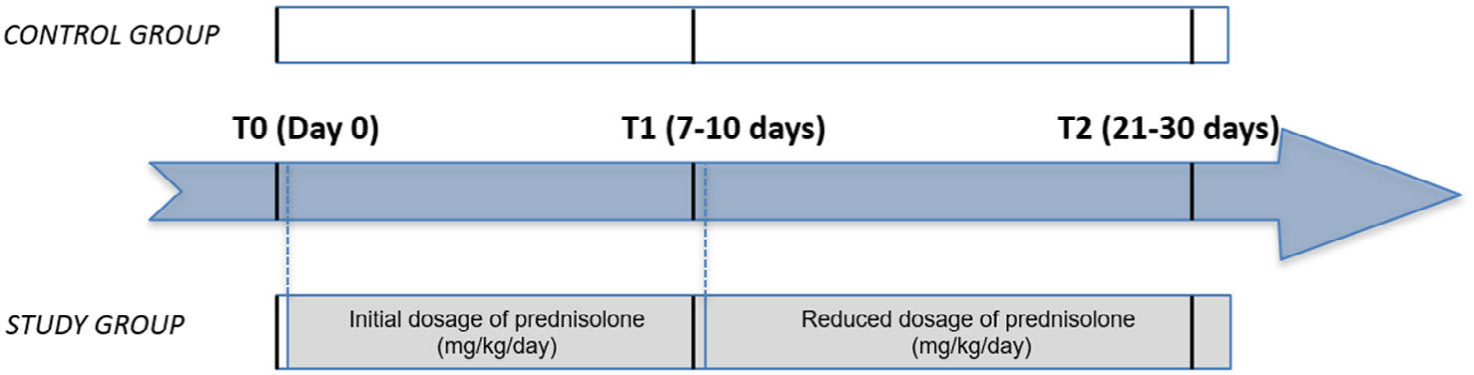
Schematic presentation of the study design. The study group (SG) comprised 17 dogs with a disease that justified the onset of prednisolone administered orally while the control group (CG) comprised 17 healthy dogs. In both groups, blood was collected at 3 time points: at diagnosis/before treatment (day 0/T0), 7 to 10 days later (T1), and 21 to 30 days later (T2) after beginning prednisolone therapy. The SG started the treatment with an initial dose of prednisolone after T0; the dose was reduced after T1 and maintained until T2. The CG did not receive any treatment

The SG comprised 17 dogs that were presented for consultation and, after obtaining a final diagnosis, were administered prednisolone for a documented medical reason. The inclusion criteria for SG included basal DGGR lipase value within the reference range (<80 U/L) and a diagnosis of a disease that justified the use of prednisolone at an initial dose of .5‐2.0 mg/kg/day PO for over 1 week, with a reduction in dose over the next 2 to 3 weeks (Figure 1).^17^ Dogs were excluded if their DGGR lipase activity values were above the reference range (80 U/L), they had received steroids over the previous 4 weeks, they were in poor clinical condition, or their life expectancy was expected to be less than the follow‐up period.^17^

The CG comprised 17 clinically apparently healthy dogs recruited from among those belonging to the medical staff and students. The dogs were clinically healthy and had no concurrent diseases. Additionally, the inclusion criteria were basal DGGR lipase values within the reference range (<80 U/L) and no use of corticosteroids over the previous 4 weeks.^17^

### 2.2 Study design

A prospective cohort study was conducted. The SG received prednisolone administered orally while the CG did not receive any treatment during the study period. In both groups, blood samples were collected at 3 time points: T0, 7 to 10 days later (T1), and 3 to 4 weeks later (T2). In the SG, these time‐points corresponded, respectively, to the time of diagnosis/before treatment and 7 to 10 and 21 to 30 days after starting prednisolone therapy (Figure 1). Serum samples were obtained by centrifugation at 2500 rpm for 10 minutes, stored at 4°C, and analyzed within the 24 hours following the blood sample collection. DGGR lipase activity was measured at all 3 time points in both groups. The analysis was performed using a kit previously validated for use in dogs (Randox DGGR lipase, LI 3837) by trained clinical pathologists per the manufacturer's guidelines at the Laboratório Prof. Doutor M. Braço Forte (Veterinary Teaching Hospital—Faculty of Veterinary Medicine—University of Lisbon).^17^

The reference interval was under 80 U/L, as previously validated in dogs and used by the testing laboratory.^17^ Recognizing a possible intraindividual variability in serum lipase activity in healthy dogs leading to values outside the reference range as well as the impossibility of ruling out occult pancreatitis in clinically healthy dogs, a 2‐fold *gray zone* (80‐160 U/L) was established.^10^

Dogs from the SG were administered prednisolone orally at an initial dose of 0.5‐2.0 mg/kg/day and then a clinically justified reduction protocol over the subsequent 21 to 30 days.^16^ The doses were reduced at T1 and the same doses were maintained in all animals until the end of the study (Figure 1). On T1, the DGGR lipase activity corresponded to the ongoing initial dose and on T2 to the ongoing reduced dose (Figure 1).

In both SG and CG, abdominal ultrasound was considered in the study protocol for dogs with increased DGGR lipase activity over 160 U/L during the study.

### 2.3 Statistical analysis

All the collected data were recorded, descriptive statistics were calculated, and figures were created using Microsoft Office Excel 2016. The statistical tests were implemented using the commercial statistical software IBM SPSS Statistics for Windows, version 26.0. *P* values <.05 were considered significant for all tests. The data were tested for normality using the Shapiro‐Wilk tests.

Statistical differences in sex and age between the SG and CG were determined. The continuous variable (age) was compared using Student's *t* test. The nominal variable (sex) was compared using Pearson's chi‐squared tests.

The DGGR lipase activity at T0, T1, and T2 was analyzed in both the SG and CG. The mean serum DGGR lipase activity at the 3 time points were then compared by repeated‐measures analysis of variance (ANOVA), which considered the Mauchly's test for sphericity and Greenhouse‐Geisser correction. Post hoc analysis was performed using the Bonferroni post hoc test.

The DGGR lipase activity at T0, T1, and T2 were compared between groups using Mann‐Whitney *U*‐tests.

To determine if a correlation existed between the DGGR lipase activity and different prednisolone doses, Spearman's rank correlation coefficient (*r*_s_) was used.

## 3 RESULTS

### 3.1 Sample population

The SG group comprised 4 (24%) female dogs aged 6 to 10 years and 13 (76%) male dogs aged 1 to 13 years. The mean age of the SG (±SD) was 6.71 ± 3.86 years. The breed distribution consisted of Yorkshire Terrier (n = 3), Labrador Retriever (n = 2), Golden Retriever (n = 1), Pit Bull Terrier (n = 1), Whippet (n = 1), Parson Terrier (n = 1), Akita Inu (n = 1), Mixed breed (n = 1), Chihuahua (n = 1), Poodle (n = 1), Spinone Italiano (n = 1), German Spitz (n = 1), Boxer (n = 1), and Miniature Pinscher (n = 1). The distribution of diseases that justified the prescription of prednisolone were chronic tracheobronchitis (n = 8), steroid‐responsive enteropathy (n = 5), ophthalmological disease (n = 2), immune‐mediated polyarthritis (n = 1), and intervertebral disc disease (n = 1). The mean presenting prednisolone dose was 0.94 ± 0.85 mg/kg/d, corresponding to the dose at T1. The dosage decreased to a mean of 0.45 ± 0.05 mg/kg after T1, corresponding to the dose at T2. All dogs from the SG had already undergone a complete blood count and basic biochemistry panel (including blood urea nitrogen, creatinine, and alanine aminotransferase) within 2 months before study inclusion and abnormalities were not detected.

The CG comprised 5 (29%) female dogs aged 7 to 10 years and 12 (71%) male dogs aged 2 to 13 years. The mean age was 7.05 ± 3.18 years. The breed distribution was Mixed breed (n = 7), Dachshund (n = 3), Yorkshire Terrier (n = 2), Shetland Sheepdog (n = 1), Jack Russell Terrier (n = 1), Great Dane (n = 1), West Highland Terrier (n = 1), and Labrador Retriever (n = 1).

The CG and SG were age and sex matched (*P* = .78 and *P* = .7, respectively).

### 3.2 DGGR lipase activity over time

**FIGURE 2:**
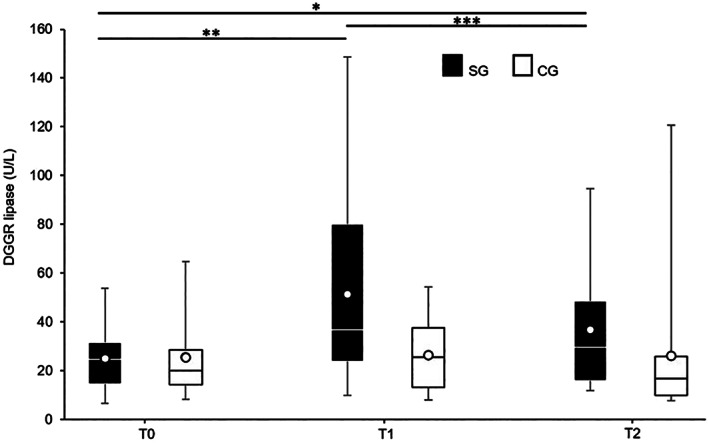
1,2‐*O*‐Dilauryl‐*rac*‐glycero glutaric acid‐(6′‐methylresorufin) ester (DGGR) lipase activity over T0, T1, and T2 in the study group (SG) and control group (CG), each comprising 17 dogs. The SG showed significant differences over time (*P* = .007) (*P = .07, **P = .02, and ***P = .02) while the CG did not (*P* = .93). The boxes indicate the lower to upper quartiles (25th‐75th percentiles) and median values with means shown as individual points. The whiskers extend to the minimum and maximum values

The median (interquartile range [IQR]) DGGR lipase activity at each time point (T0, T1, and T2) in the SG was 24.74 (14.45‐31.48), 36.82 (23.8‐80.16), and 29.52 (15.91‐48.48) U/L, respectively (Figure 2). A statistically significant effect of prednisolone on DGGR lipase activity was observed (*F*[1.119, 17.901] = 8.903, *P* = .007). The DGGR lipase activity increased from T0 to T1 and decreased from T1 to T2, both with statistically significant differences (*P* = .02 and *P* = .02, respectively). The difference was not statistically significant from T0 to T2 (*P* = .07).

The median DGGR lipase activity at each time point (T0, T1, and T2) in the CG was 19.91 (14.12‐28.5), 25.46 (13.25‐37.64), and 16.84 (9.78‐25.8) U/L, respectively (Figure 2). The mean serum DGGR lipase activity did not differ significantly over time (*F*[1.311, 20.980] = .025, *P* = .93).

### 3.3 Comparisons of DGGR lipase activity between groups

At T0, the difference in the DGGR lipase activity in the SG was not statistically significant compared to the activity in the CG (*P* = .71). T1 also did not differ significantly between the SG and CG (*P* = .09). T2 differed significantly between the SG and CG (*P* = .05).

### 3.4 Correlations between DGGR lipase activity and prednisolone doses

**FIGURE 3:**
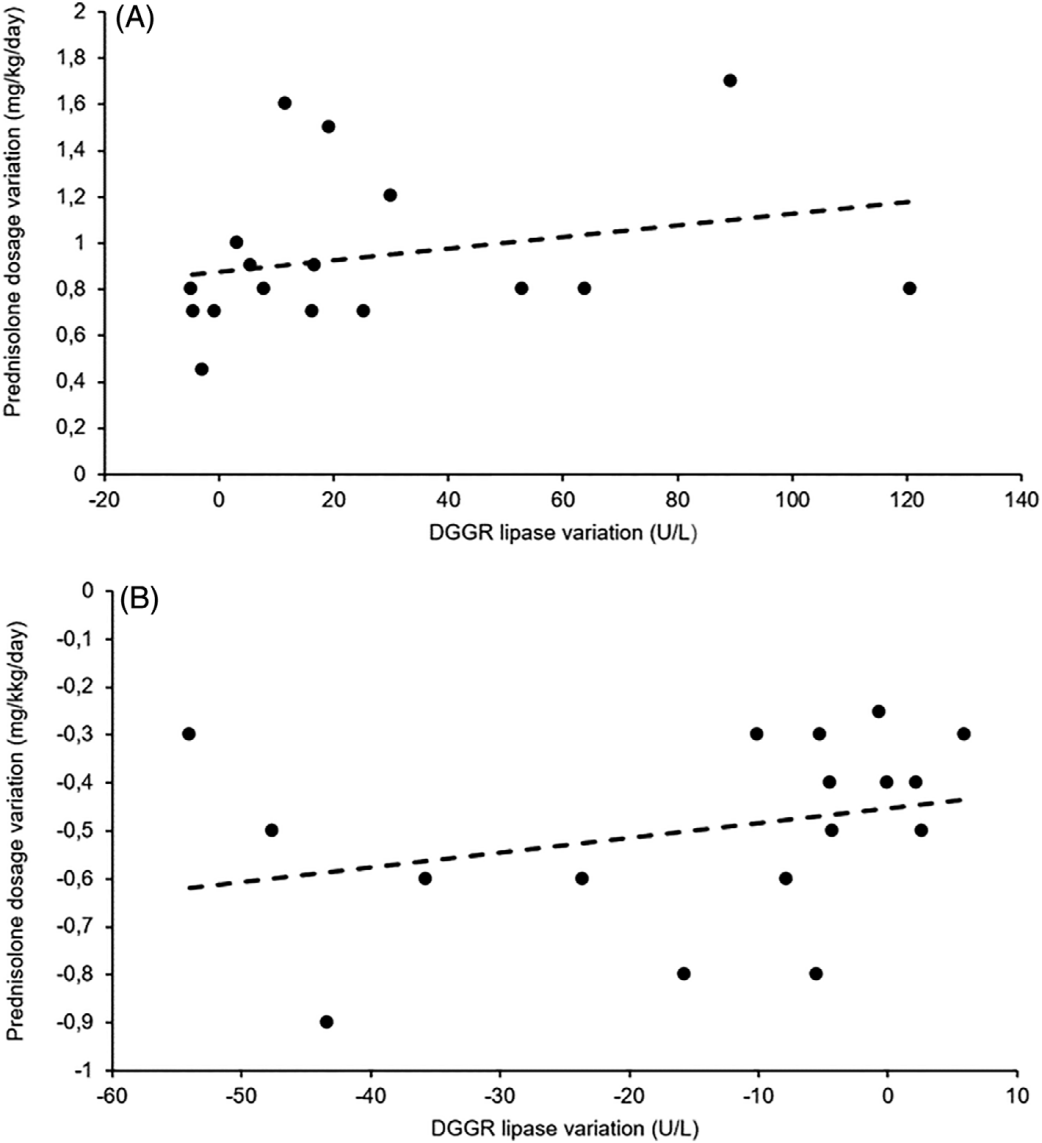
Graphs of the variations in 1,2‐*O*‐dilauryl‐*rac*‐glycero glutaric acid‐(6′‐methylresorufin) ester (DGGR) lipase activity and variations in prednisolone doses of the 17 dogs from the study group (SG) from T0 to T1 (A) and from T1 to T2 (B). The variations in DGGR lipase activity were not significantly correlated with the variations in prednisolone doses at T0‐T1 (*r*_s_ = .371; *P* = .14) and T1‐T2 (*r*_s_ = .390; *P* = .12). At T0‐T1 and T1‐T2, the equations for the best‐fit lines were *y* = 0.0025*x* + 0.8779 and *y* = 0.0031*x* − 0.4521, respectively

The variations in DGGR lipase activity were not significantly correlated with the variations in prednisolone doses at T0‐T1 (*r*_s_ = 0.371, *P* = .14) (Figure 3A) or T1‐T2 (*r*_s_ = 0.390, *P* = .12) (Figure 3B).

## 4 DISCUSSION

The results of this study showed that DGGR lipase activity can be affected by prednisolone therapy in dogs. However, these effects did not appear to be clinically relevant as the values remained below the upper normal limit (<160 U/L).

Dogs with hyperadrenocorticism can have DGGR lipase activity above the reference interval.^11^, ^12^ As glucocorticoids are commonly prescribed in veterinary medicine, this study clarified that DGGR lipase activity is also affected by prednisolone therapy.^16^

The CG and SG were age and sex matched to reduce variable differences apart from prednisolone treatment. Therefore, it was possible to evaluate and compare the differences in DGGR lipase activity over time between the SG and CG and within each group.

Regarding the comparison of DGGR lipase values between SG and CG, no statistically significant difference in DGGR lipase activity was observed at the time point before starting prednisolone therapy (T0). Then, analyzing DGGR lipase variation from that point showed that SG and CG evolved differently.

The CG did not show statistically significant differences over time. The observed intraindividual variability reinforced the need for a larger number of dogs to reduce the variability and increase the statistical power of the present study. Intraindividual biological variation could also explain the higher values over time in the CG, such as 120 U/L at T2 (Figure 2).

In dogs treated with prednisolone, a variation in DGGR lipase was observed, characterized by an initial increase from T0 to T1 and subsequent decrease from T1 to T2. Prednisolone was administered from T0 to T1 and decreased from T1 to T2. Although the SG evolved differently, no significant difference was observed when comparing T2 to T0 in this group. However, DGGR lipase activity was significantly higher in the SG than those in the CG at this time point. Beyond the above‐mentioned intraindividual biological variations, these results indicated that prednisolone therapy appeared to have a residual but clinically negligible effect when administered at lower doses and despite cumulative therapy.

The variation over time was not correlated with the variation in prednisolone dosage. Previous studies indicated that prednisolone dose might be important in determining its effect on pancreatic tissue and, consequently, cPLI.^14^ Although a study using prednisone at 2.2 mg/kg/day PO showed no effect on cPLI, another tested prednisolone at 2.0 to 2.2 mg/kg/day PO and reported cPLI increases above the reference range.^13^, ^15^ Compared to previous results, the nonexistent correlation between DGGR lipase activity and prednisolone dose in this study might be related to differences in bioavailability between prednisolone and prednisone.^18^ Another possible explanation for this fact is the small sample size of the groups, which might have contributed to the observed correlation or there might have been no correlation.

Despite the variation observed in the SG, the median values did not exceed the upper limit of the considered reference interval (80 U/L). The maximum recorded activity, 148.7 U/L, did not exceed the upper limit of the *gray zone* (80‐160 U/L). As previously mentioned, the 2‐fold *gray zone* was based on the findings of another study to consider possible intraindividual variability in serum lipase activity in healthy dogs as well as the inability to rule out transient/occult mild pancreatitis in clinically healthy dogs.^10^ Therefore, no dogs reached the defined clinically relevant cutoff for pancreatitis.

From a pathophysiological perspective, it is unclear what caused the serum DGGR lipase activity to increase over time in dogs administered prednisolone treatment. The main possible explanations rely on the evolution of occult and subclinical pancreatitis during the study period or due to extrapancreatic factors that can induce increased DGGR lipase activity.

No values above 160 U/L were observed in this study, the upper limit of the *gray zone*, and therefore abdominal ultrasound was not performed in any of these dogs.^10^ Moreover, the correlation between abdominal ultrasound findings and the clinical diagnosis of pancreatitis is moderate.^19^ The different sensitivities of abdominal ultrasound for the diagnosis of pancreatitis could reflect various factors such as the suspicion level, operator skills, equipment, and lesion severity.^20^ Moreover, the sensitivity and specificity for the detection of clinical pancreatitis vary greatly among diagnostic criteria, with no significant associations between the ultrasonographic assessment of pancreatitis severity and clinical severity.^19^ Furthermore, the agreement between ultrasonographic diagnosis of pancreatitis and DGGR lipase activity is poor, even though it improves with higher cutoff values.^10^ Although abdominal ultrasound might have been helpful to evaluate the pancreatic region and to exclude other differential diagnoses, it was considered unnecessary to perform as none of the dogs in the present study had clinical signs of pancreatitis such as vomiting, diarrhea, or abdominal pain.^20^ Of the 17 dogs in the SG, 5 were diagnosed with steroid‐responsive enteropathy. These dogs had a previous history of diarrhea, which could be compatible with pancreatic disease. However, fulfilling the inclusion criterion, all of these animals had DGGR lipase activity within the reference range (<80 U/L), thus meeting the study objective. Furthermore, during the previous clinical investigation, abdominal ultrasound examinations had been performed in 4 of these 5 dogs, in which the pancreatic region did not show relevant ultrasonographic findings. Moreover, the signs of gastrointestinal disease made these 5 animals a subgroup, which allowed observation of DGGR lipase activity variation in dogs with gastrointestinal disease receiving prednisolone.

As glucocorticoids cause pancreatitis in small animals,^16^ occult pancreatitis in dogs receiving prednisolone therapy could have occurred. However, the relationship between pancreatitis and glucocorticoids was mainly based on sporadic cases with other associated risk factors. Despite this controversy, corticosteroids are not considered a risk factor for pancreatitis.^16^ Moreover, a study reporting a significant increase in serum cPLI concentration during prednisolone treatment did not observe pancreatic histological abnormalities before and after prednisolone administration.^14^ Limitations of the previous studies included a noncomplete histological examination, only 3 weeks of observation, and a small sample size. Therefore, glucocorticoids as a cause of pancreatitis has been largely dismissed in dogs and cats.^16^

Apart from occult pancreatitis, other reasons might explain the increase in DGGR lipase activity, including increased pancreatic lipase synthesis, increased cellular permeability to this enzyme, or a decreased rate of its renal clearance.^14^ The effect of renal disease on lipase activity is controversial. ^21^, ^22^, ^23^, ^24^ In the present study, the animals in the SG did not have azotemia and did not have related clinical signs over the study period. Although the glomerular filtration rate was not evaluated, it was unlikely to have decreased, particularly in dogs receiving prednisolone therapy. Lipase production from extrapancreatic tissues was also possible. In contrast to previous studies, pancreatic lipase messenger ribonucleic acid (mRNA) expression was not assessed in any tissue.^14^ Furthermore, DGGR lipase is not specific to the pancreas; thus, nonpancreatic production of lipase cannot be ruled out in this study.^25^ In addition, glucocorticoids play a role in lipid metabolism by stimulating hormone‐sensitive lipase and inhibiting lipoprotein lipase.^26^ Hormone‐sensitive lipase promotes adipocyte lipolysis, while lipoprotein lipase promotes fat uptake into adipocytes.^27^ In addition, hypertriglyceridemia associated with hyperadrenocorticism might be due to the stimulation of hormone‐sensitive lipase.^26^ Therefore, stimulation of hormone‐sensitive lipase activity supports the possibility of a nonpancreatic origin for lipase increase over coticotherapy.

This study had some limitations. First, the absence of histopathological pancreatic examination impaired the assessment of concurrent tissue changes that might occur with prednisolone therapy. As histopathology is the current gold standard for the diagnosis of pancreatitis, it would have been useful to have investigated tissue changes. Although the relevance of histopathology was recently confirmed, this procedure is not preferable because of its invasiveness, the possibility of insufficient results related to the nonuniform lesion distribution, and for ethical reasons.^28^, ^29^ Therefore, to address these limitations, recent studies have used clinical diagnosis as a gold standard for pancreatitis.^19^ Thus, although pancreatitis cannot be completely ruled out even if biopsy is performed, it would not be highly sensitive in asymptomatic dogs because of the possibility of occult inflammation associated with localized histologic lesions.^29^

Obesity and hyperlipidemia are considered risk factors for pancreatitis in dogs.^20^ It would have been useful to explore the possible correlation between triglycerides and cholesterol serum levels, body condition scores, and DGGR lipase activity in both groups.^20^ This is another limitation of the present study.

Being a colorimetric method, the absence of mild hemolysis or lipemia, which is likely to affect DGGR measurement, was not assured in all samples from both groups. Although recommended, fasting was not always performed depending on owner compliance which, consequently, might have affected lipemia. However, no sample showed gross lipemia or hemolysis and minimal changes were observed in the presence of high concentrations of lipid and hemoglobin for lipase activity in the DGGR assay.^9^ Additionally, hemolysis does not result in significant changes in DGGR lipase activity and nor does 12 months of sample storage at −80°C.^4^

Another limitation was the fact that the oral administration of prednisolone was owner dependent. Compliance was, therefore, a strong constraining factor and, since it was not possible to assure dosing, this was a potential limitation of the present study. Nonetheless, as dogs improved from the medical conditions that justified prednisolone therapy, it is unlikely that the therapeutic schedule was dismissed.

The dosage range in this study was also a limitation as it was case dependent and it was not possible to standardize or administer a narrower range. Furthermore, the dosage range was not maintained over the study period because of medical reasons. Despite the nature of this clinical study, the prescribed dosages were applied to each animal depending on the underlying disease and individual glucocorticoid sensitivity.^16^ After the first treatment, the dosage was reduced to the lowest necessary for control of clinical signs.^16^

Finally, the number of dogs used in this study was small and more dogs would be needed to increase the statistical power. Regardless of the development of pancreatitis, the underlying disorder itself, diversity in dosage, age, and sex could have contributed to the diversity of DGGR results among the dogs.

## 5 CONCLUSION

While prednisolone can affect DGGR lipase activity, this effect was not clinically important suggesting that high DGGR lipase activity in a dog administered prednisolone merits consideration of pancreatitis.

## CONFLICT OF INTEREST DECLARATION

Authors declare no conflict of interest.

## OFF‐LABEL ANTIMICROBIAL DECLARATION

Authors declare no off‐label use of antimicrobials.

## INSTITUTIONAL ANIMAL CARE AND USE COMMITTEE (IACUC) OR OTHER APPROVAL DECLARATION

Approved by the local welfare and ethical committee Comissão de Ética e Bem‐Estar Animal (CEBEA) ‐ Faculdade de Medicina Veterinária ‐ Universidade de Lisboa.

## HUMAN ETHICS APPROVAL DECLARATION

Authors declare human ethics approval was not needed for this study.
